# Supplementary material for: Multicohort Analysis Identifies Monocyte Gene Signatures to Accurately Monitor Subset-Specific Changes in Human Diseases
Source: Front Immunol. 2021 May 14;12:659255. doi: 10.3389/fimmu.2021.659255 (PMC8160521; doi:10.3389/fimmu.2021.659255)
Supplement: Supplementary file 1 [file DataSheet_1.pdf]

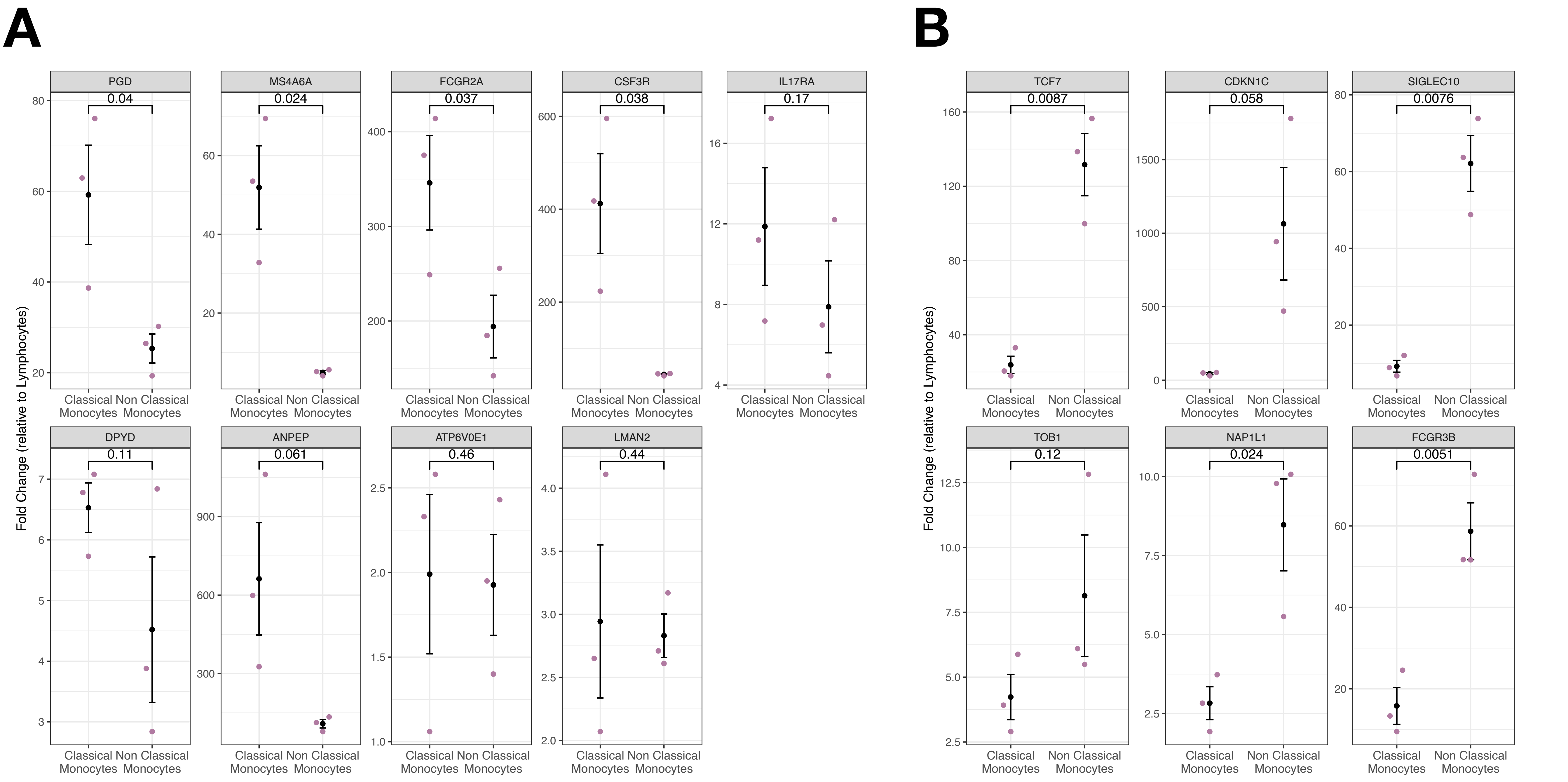

Supplemental Figure 1: **Independent qPCR validation of subset-specific signatures:** (a) Dotplots representing classical signature genes fold change measured by qPCR on sorted classical and non classical monocytes from healthy human samples. Significance was estimated by t-test (b) Same as in (a) but for non classical signature genes.

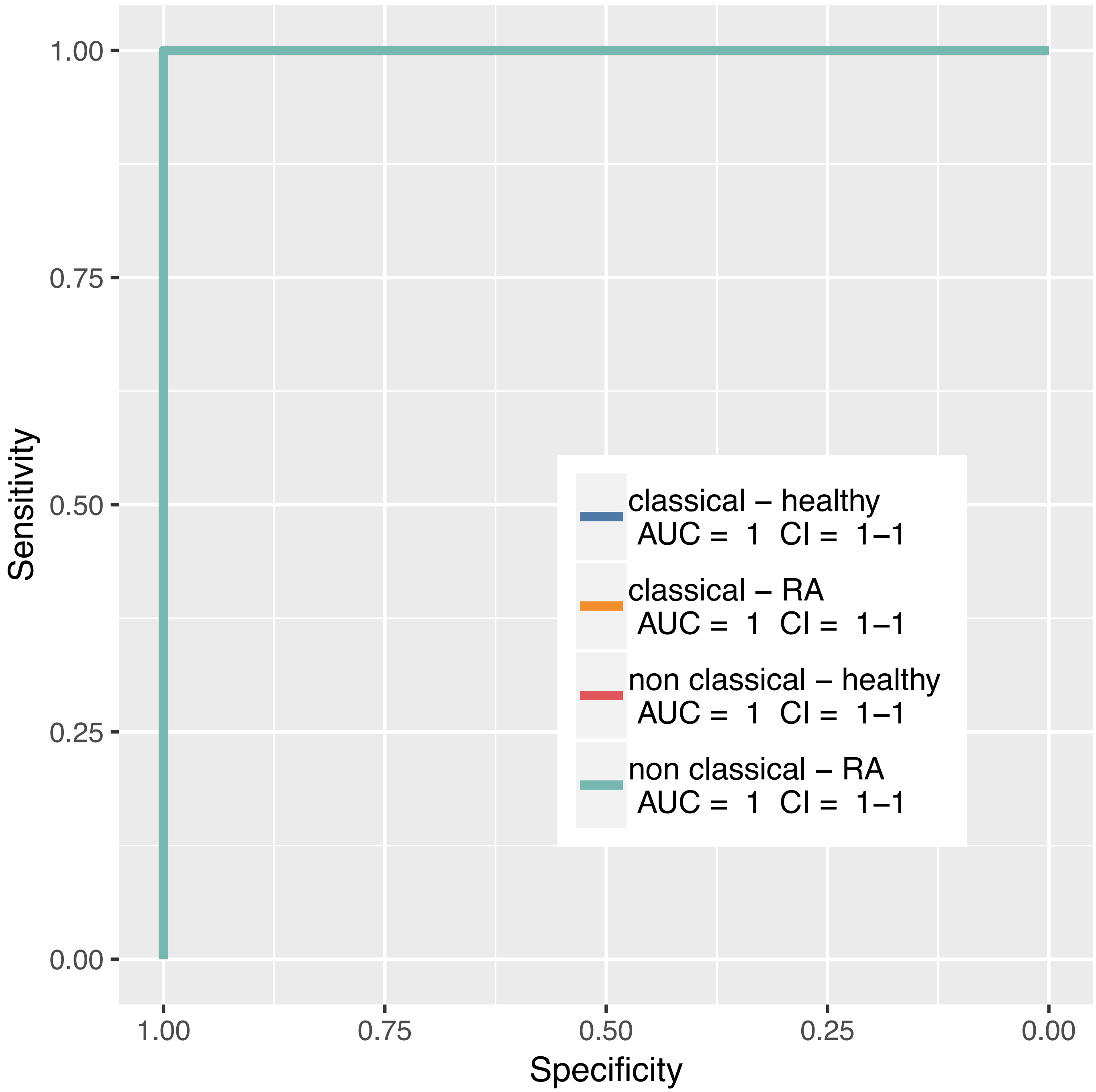

Supplemental Figure 2: **Accuracy in subset classification on independent validation cohort:** ROC curves depicting accuracy of subset classification by corresponding gene expression signature across healthy and RA-affected samples.

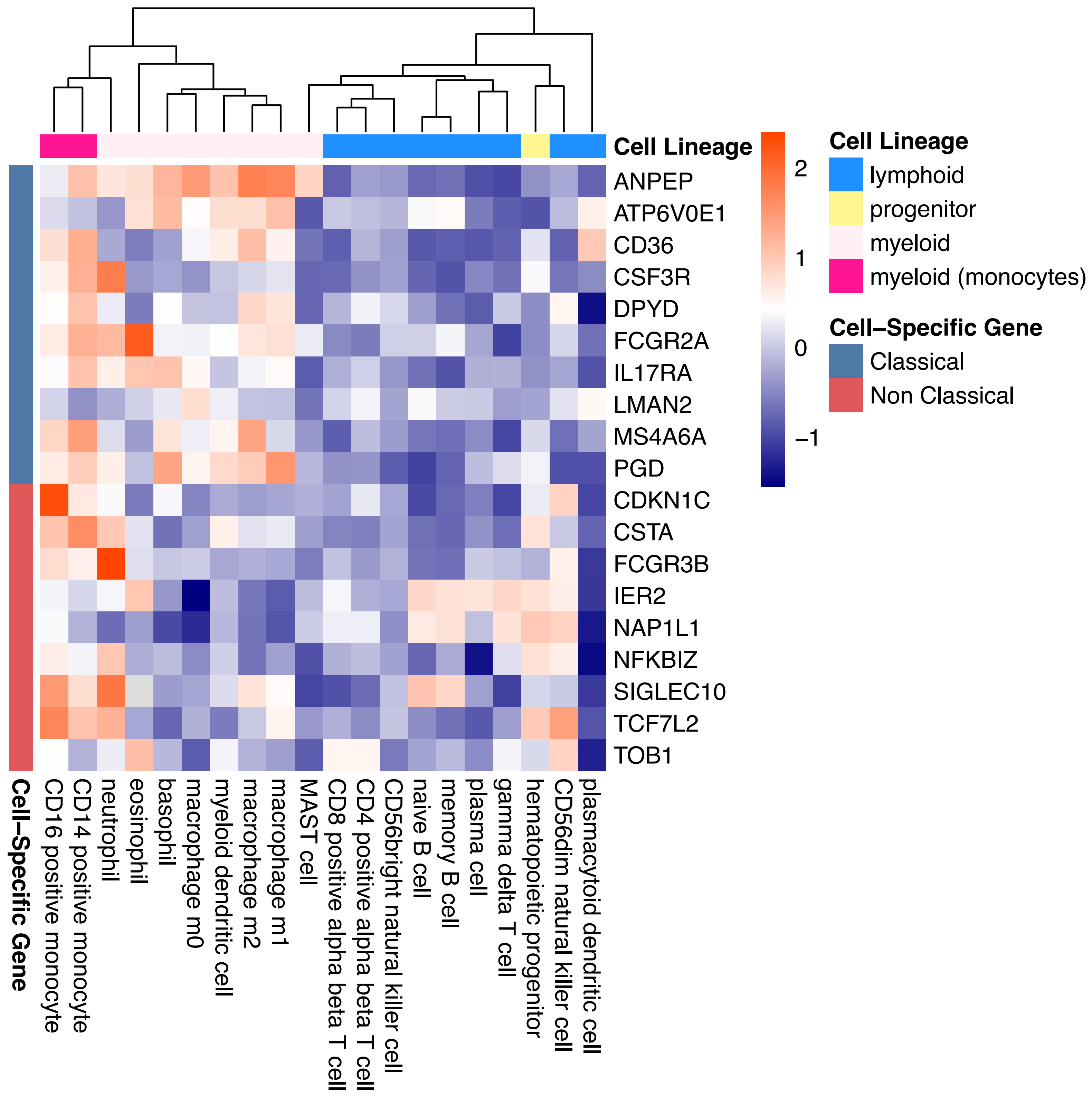

Supplemental Figure 3: **Monocyte signatures are specific across all immune cells:** Heat-map displaying gene expression effect sizes of monocyte subset-specific signature genes across 20 human immune cells.

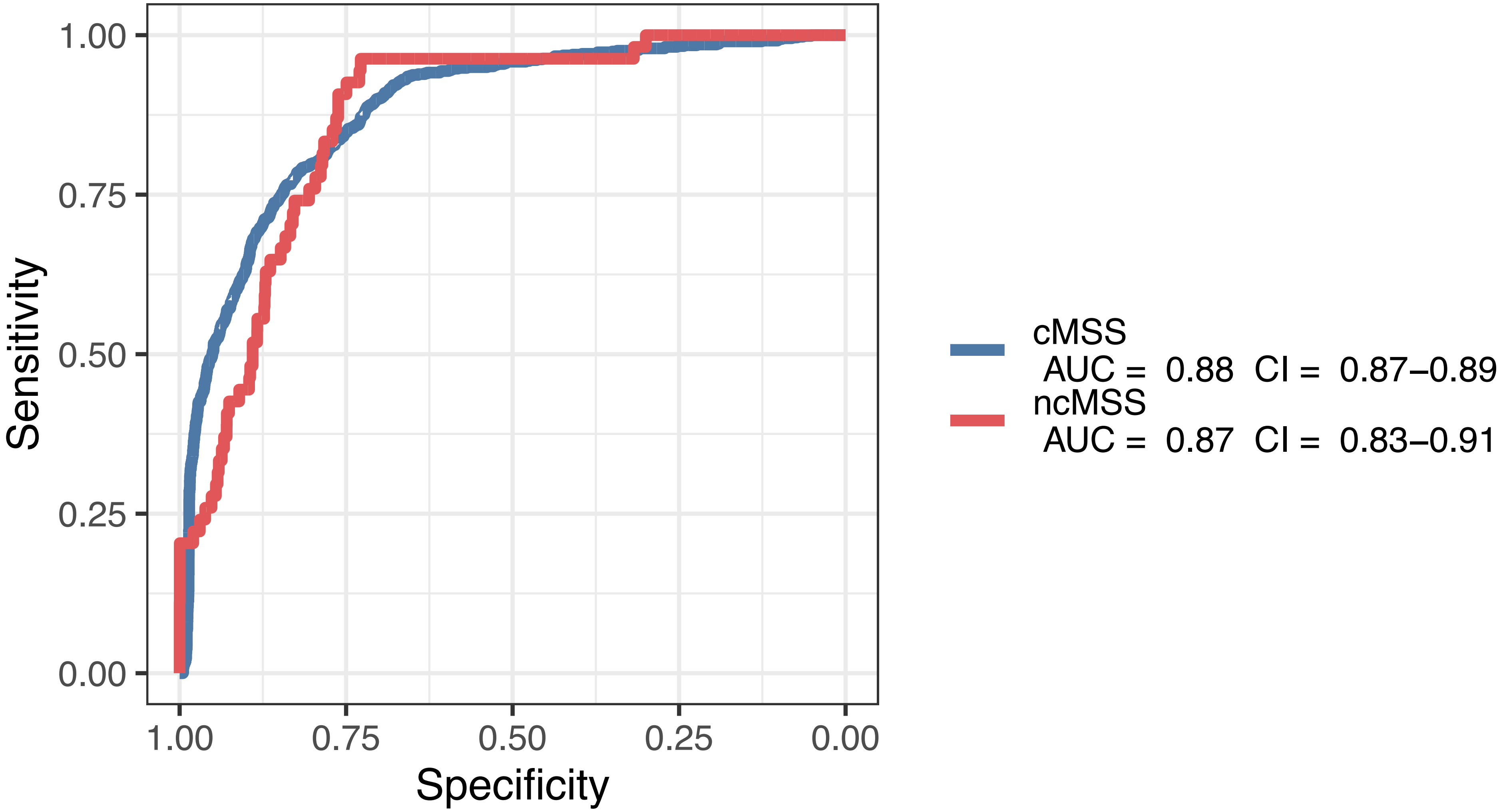

Supplemental Figure 4: **Monocyte signatures are specific across all immune cells:** ROC curves depicting accuracy of subset classification by corresponding gene expression signature across 6160 transcriptomes profiling sorted human immune cells

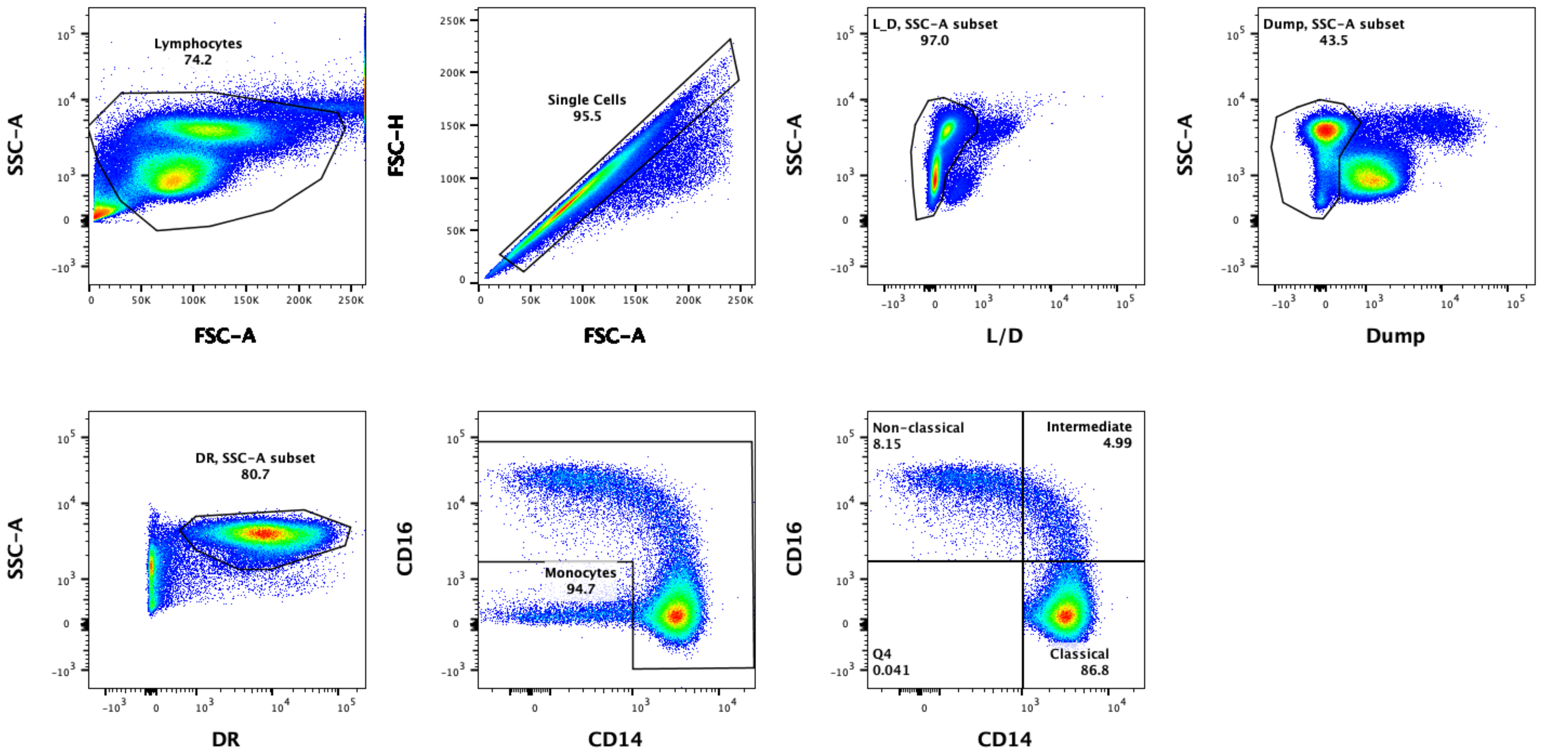

Supplemental Figure 5: **Flow cytometry gating strategy to identify monocyte subsets in peripheral mononuclear cells (PBMCs):** PBMCs (labeled as "Lymphocytes") were gated on a side scatter (SSC) v. forward scatter (FSC) plot, followed by singlet gating based on FSC-H (Height) v. FSC-A (Area). Live cells were determined as negative for LIVE/DEAD (L/D) Aqua staining. T cells (CD3), B cells (CD19), NK cells (CD56), dendritic cells (CD1c and CD141) and neutrophils (CD66b) were excluded as a dump channel. HLA-DR positive cells were selected and plotted on a CD16 vs CD14 plot to identify total monocytes. Gates for classical (CD14+CD16-), intermediate (CD14+CD16+), and nonclassical (CD14-CD16+) subsets are based on Fluorescence minus one (FMO) controls.

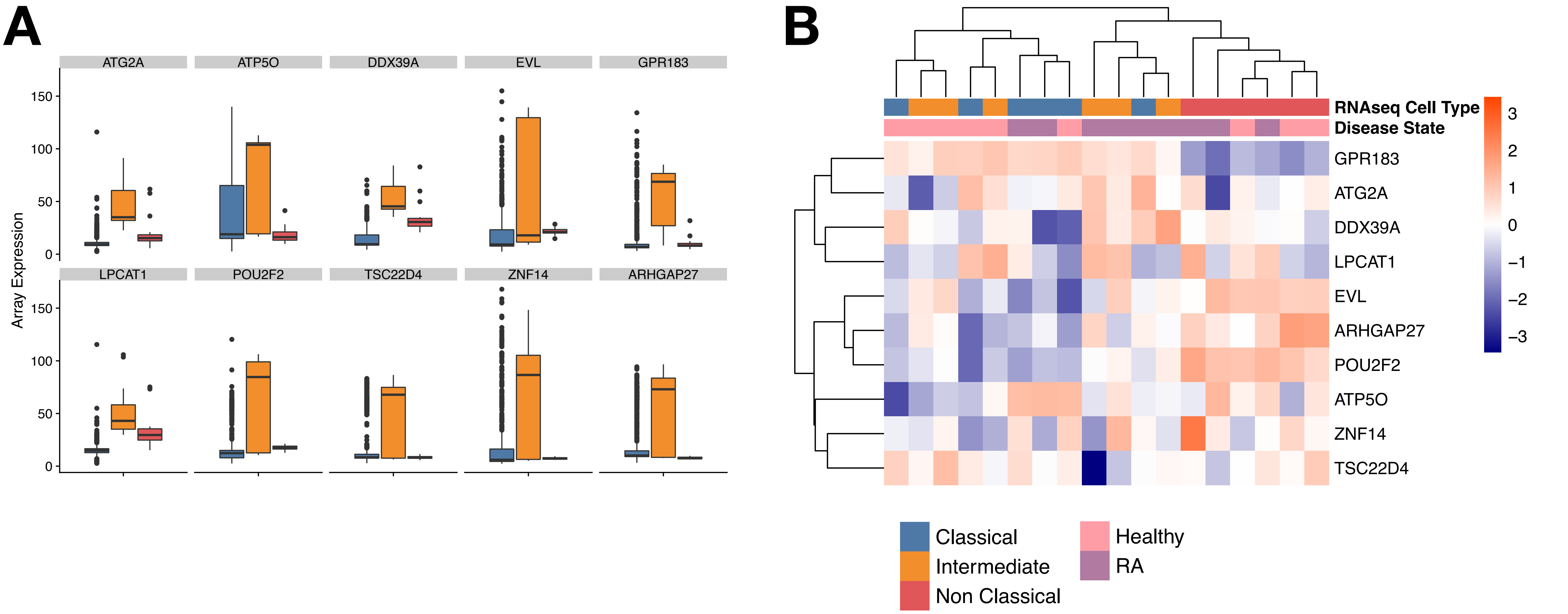

Supplemental Figure 6: **Intermediate monocytes are not distinguishable by our transcriptional signatures:** (a) Selection of an intermediate subset gene signature in the discovery cohort. (b) Heat-map displaying the expression of the intermediate subset genes in the RNA-seq validation dataset.
